# Identification of the Extracytoplasmic Function σ Factor σ^P^ Regulon in Bacillus thuringiensis

**DOI:** 10.1128/msphere.00967-21

**Published:** 2022-01-26

**Authors:** Theresa D. Ho, Kelsie M. Nauta, Emma K. Luhmann, Lilliana Radoshevich, Craig D. Ellermeier

**Affiliations:** a Department of Microbiology and Immunology, Carver College of Medicine, University of Iowa, Iowa City, Iowa, USA; b Graduate Program in Genetics, University of Iowa, Iowa City, Iowa, USA; University of Rochester

**Keywords:** cell envelope, stress response, signal transduction, gene expression, sigma factors

## Abstract

Bacillus thuringiensis and other members of the Bacillus cereus family are resistant to many β-lactams. Resistance is dependent upon the extracytoplasmic function sigma factor σ^P^. We used label-free quantitative proteomics to identify proteins whose expression was dependent upon σ^P^. We compared the protein profiles of strains which either lacked σ^P^ or overexpressed σ^P^. We identified 8 members of the σ^P^ regulon which included four β-lactamases as well as three penicillin-binding proteins (PBPs). Using transcriptional reporters, we confirmed that these genes are induced by β-lactams in a σ^P^-dependent manner. These genes were deleted individually or in various combinations to determine their role in resistance to a subset of β-lactams, including ampicillin, methicillin, cephalexin, and cephalothin. We found that different combinations of β-lactamases and PBPs are involved in resistance to different β-lactams. Our data show that B. thuringiensis utilizes a suite of enzymes to protect itself from β-lactam antibiotics.

**IMPORTANCE** Antimicrobial resistance is major concern for public health. β-Lactams remain an important treatment option for many diseases. However, the spread of β-lactam resistance continues to rise. Many pathogens acquire antibiotic resistance from environmental bacteria. Thus, understanding β-lactam resistance in environmental strains may provide insights into additional mechanisms of antibiotic resistance. Here, we describe how a single regulatory system, σ^P^, in B. thuringiensis controls expression of multiple genes involved in resistance to β-lactams. Our findings indicate that some of these genes are partially redundant. Our data also suggest that the large number of genes controlled by σ^P^ results in increased resistance to a wider range of β-lactam classes than any single gene could provide.

## INTRODUCTION

The bacterial cell wall is made up of peptidoglycan, a unique structure that provides the cell rigidity and shape. Peptidoglycan also protects bacteria from lysis due to turgor pressure. The peptidoglycan is a polymer of repeating *N*-acetylmuramic acid (NAM) and *N*-acetylglucosamine (NAG) strands cross-linked between pentapeptide side chains (1–4). The precursors for peptidoglycan synthesis are synthesized in the cytoplasm, linked to lipid carriers to form lipid II, and flipped across the cytoplasmic membrane. Once on the extracellular side of the membrane, the NAG-NAM pentapeptide precursor is added to the growing peptidoglycan chain by peptidoglycan glycosyltransferases (2, 3). The peptide side chains are then cross-linked by transpeptidases (2, 3).

In most bacteria, penicillin-binding proteins (PBPs) perform a transpeptidase reaction to cross-link the second d-alanine (d-Ala) of one donor side chain to the *m*-diaminopimelic acid (mDap) or lysine (Lys) at position 3 of the acceptor side chain (2, 3). PBPs fall into one of three broad classes. Class A PBPs (aPBPs) possess both transglycosylase and transpeptidase activities (2, 3). Class B PBPs (bPBPs) function as transpeptidases but lack transglycosylase activity (2, 3). In most cases, bPBPs interact with a SEDS (shape, elongation, division, and sporulation) family of proteins which provide the transglycosylase activity (2, 4). Unlike class A and B PBPs, which have highly homologous transpeptidase and substrate-binding domains, class C PBPs have lower molecular weights and are structurally distinct. Class C PBPs are often carboxypeptidases that remove the d-Ala–d-Ala from the peptide side chains of peptidoglycan (2, 3, 5).

Peptidoglycan is essential for bacterial growth and survival. Thus, inhibition of peptidoglycan synthesis is often lethal. β-Lactams are a class of antibiotic which kill cells by inhibiting peptidoglycan cross-linking (6, 7). β-Lactams resemble the terminal d-Ala–d-Ala of the peptidoglycan pentapeptide side chain (8, 9). This resemblance leads to β-lactams being recognized by PBPs (9, 10). β-Lactams become covalently attached to PBPs when the active-site serine of a PBP performs nucleophilic attack on the β-lactams and forms a covalent bond (10, 11). Thus, β-lactams act as suicide inhibitors of PBPs.

There are four main classes of β-lactams (10, 12–16). All four families have a four-member β-lactam ring (10, 14). These families include the following: the penicillins, which have a β-lactam ring fused to a thiazolidine ring; the cephalosporins, which have a β-lactam ring fused to a six-membered ring; the carbapenems, which have a β-lactam ring fused to a five-membered pyrroline; and the monobactams, which contain only the four-membered β-lactam ring (10, 16).

Each of these classes of antibiotics exists naturally, and thus, unsurprisingly, bacteria have evolved a variety of β-lactam resistance mechanisms. In some Gram-negative bacteria, resistance is mediated by decreased access of β-lactams to the PBPs due to decreased outer membrane permeability or increased export (11). Another mechanism of resistance is production of a modified PBP which has decreased binding to the β-lactam yet retains the ability to form cross-links between pentapeptide side chains (11). Modified PBPs with increased β-lactam resistance have been identified in both Gram-positive and Gram-negative bacteria (11, 17–19). Both aPBPs and bPBPs have been shown to be capable of resisting β-lactams in multiple organisms due to decreased β-lactam affinity (11).

The most common mechanism is mediated by β-lactamases, which degrade the antibiotic itself. β-Lactamases hydrolyze the amide bond and destroy the β-lactam ring. There are four classes of β-lactamases, A to D. Classes A, C, and D are structurally similar and require an active-site serine for β-lactamase activity. In this way, classes A, C, and D resemble PBPs, but unlike PBPs, they are able to hydrolyze the amide bond, leading to destruction of the β-lactam ring (10, 12). Class B β-lactamases are structurally distinct metallo-β-lactamases that utilize Zn^2+^ ions to coordinate their active site (10, 12). There have been over 4,000 different β-lactamases identified (10, 12).

Bacillus thuringiensis, Bacillus cereus, and Bacillus anthracis are closely related soil organisms that must compete with other bacteria in the environment. Members of these species are also important human, animal, and insect pathogens. Each of these species encodes the σ^P^ regulatory system, which senses and protects against β-lactams (20–22). The ECF sigma factor σ^P^ is encoded by the *sigP* gene, just upstream of its cognate anti-sigma factor gene *rsiP*. Previous work has shown that the σ^P^ regulatory system is activated by a group of β-lactams and protects against killing by some β-lactams (23, 24). In this work, we define multiple mechanisms of β-lactam resistance controlled by σ^P^ and distinguish the relative contributions of these resistance mechanisms to β-lactam resistance in B. thuringiensis.

## RESULTS

### Identification of σ^P^-regulated genes.

B. anthracis, B. cereus, and B. thuringiensis are highly related species that exhibit a high degree of resistance to β-lactam antibiotics. These three species harbor the *sigP-rsiP* operon, which encodes the ECF sigma factor σ^P^ gene and its cognate anti-sigma factor RsiP (20). Previous work found that σ^P^ is required for resistance to several β-lactams (20, 23). It was also found that σ^P^ is required for expression of genes encoding β-lactamases (20, 25). To further characterize the σ^P^ regulon, we used mass spectrometry to examine the protein profiles of different mutants in the σ^P^ signaling pathway. In previous work, we isolated a mutation of *rsiP* (*rsiP^1-80^*) which produced a truncated RsiP and results in constitutive σ^P^. The expression of *sigP* in the *rsiP^1-80^* mutant was 200-fold greater than *sigP* expression in the wild type (23). We compared the *rsiP^1-80^* mutant to a Δ*sigP-rsiP* mutant that lacks σ^P^ in order to ensure the greatest differences between strains using whole-cell proteomics (23). We grew these bacteria to a mid-log optical density at 600 nm (OD_600_) of 0.8, collected the cell pellets from biological triplicates of each strain, and quantified proteins using label-free mass spectrometry. We identified a total of 895 proteins across all samples using MaxQuant and were able to reliably quantify 327 of these proteins, meaning that unique peptides from these proteins were present in a minimum of three samples. Following log2 transformation of the data using Perseus (26), we identified 10 proteins that were decreased in abundance by greater than 2.3 logs in the Δ*sigP-rsiP* cells relative to *rsiP^1-80^* (Table 1). In total, these 10 proteins include 4 putative β-lactamases, a penicillin-binding protein, a peptidoglycan carboxy peptidase, and a putative peptidoglycan hydrolase, 6 of which had not been previously identified as regulated by σ^P^. Previous work showed that the *sigP-rsiP* operon is transcribed in a σ^P^-dependent manner (20, 23), neither of which were detected in our proteomics screen. This suggests that our proteomic screen was not exhaustive; however, we were able to identify several putative new members of the σ^P^ regulon.

**TABLE 1:** Protein fold differences

Fold change,^*a*^ Δ*sigP-rsiP/rsiP^1-80^*	HD73 name	New name	Predicted function
5.37	HD73_3486	Bla1	β-Lactamase
5.27	HD73_3491	PbpN	Serine-type d-Ala–d-Ala carboxypeptidase
5.27	HD73_2786	Bla3	β-Lactamase
4.60	HD73_3687	Bla2	β-Lactamase
3.81	HD73_2718		GDSL-like lipase/acylhydrolase family protein
3.64	HD73_2729	Bla4	β-Lactamase
2.80	HD73_1600	DltC	d-Alanine–poly(phosphoribitol) ligase subunit
2.68	HD73_2717	DacC	Serine-type d-Ala–d-Ala carboxypeptidase
2.45	HD73_1021		*N*-Acetylmuramoyl-l-alanine amidase family protein
2.36	HD73_0522		Uncharacterized protein

a *P* < 0.01.

### Confirmation of proteomic analysis.

The proteins of the putative σ^P^ regulon could be classified into 3 main categories: penicillin-binding proteins, β-lactamases, and other proteins. To determine if the expression of these genes was regulated in a σ^P^-dependent manner, we cloned the putative promoter regions (200 to 500 bp upstream of each open reading frame) in front of a *lacZ* reporter and integrated each promoter *lacZ* fusion into the *thrC* gene (23). Each transcriptional fusion was introduced into wild-type and Δ*sigP-rsiP* strain backgrounds to determine if expression was σ^P^ dependent.

**FIG 1:**
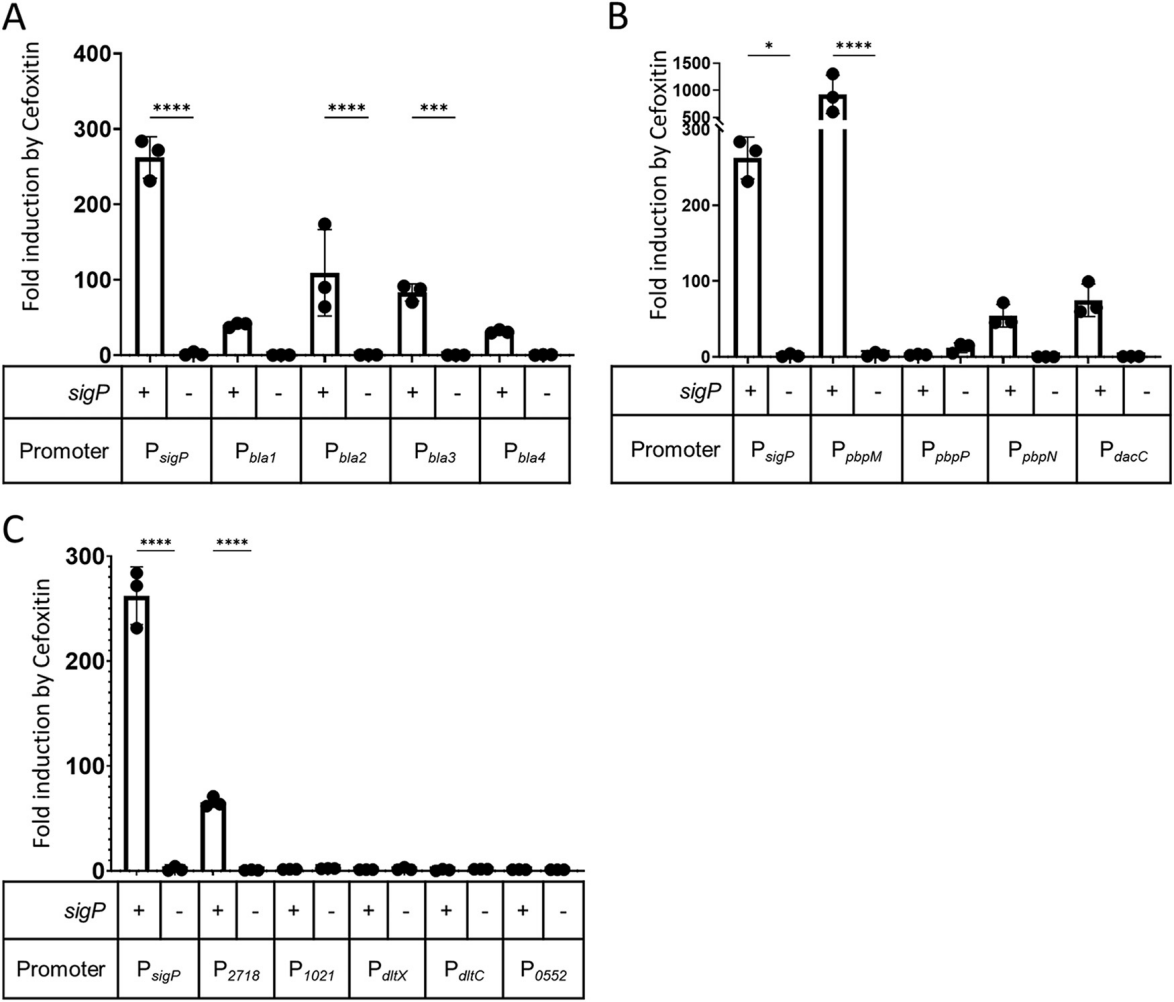
Expression of putative σ^P^-regulated genes. (A) Expression of putative β-lactamase genes. Wild-type B. thuringiensis strains with the transcriptional fusion P*_sigP_*-*lacZ* (THE2549), P*_bla1_*-*lacZ* (EBT228), P*_bla2_*-*lacZ* (EBT867), P*_bla3_*-*lacZ* (EBT322), or P*_bla4_*-*lacZ* (EBT869) or B. thuringiensis *sigP-rsiP* deletion strains containing the transcriptional fusion P*_sigP_*-*lacZ* (EBT240), P*_bla1_*-*lacZ* (EBT479), P*_bla2_*-*lacZ* (EBT868), P*_bla3_*-*lacZ* (EBT324), or P*_bla4_*-*lacZ* (EBT870) were grown in the presence and absence of the inducer cefoxitin as described in Materials and Methods. (B) Expression of putative penicillin-binding protein genes. Wild-type B. thuringiensis strains with the transcriptional fusion P*_sigP_*-*lacZ* (THE2549), P*_pbpM_*-*lacZ* (THE2548), P*_pbpP_*-*lacZ* (EBT234), P*_pbpN_*-*lacZ* (THE2547), or P*_dacC_*-*lacZ* (EBT224) or B. thuringiensis *sigP-rsiP* deletion strains containing the transcriptional fusion P*_sigP_*-*lacZ* (EBT240), P*_pbpM_*-*lacZ* (EBT476), P*_pbpP_*-*lacZ* (EBT477), P*_pbpN_*-*lacZ* (EBT478), or P*_dacC_*-*lacZ* (EBT480) were grown in the presence and absence of the inducer cefoxitin as described in Materials and Methods. (C) Expression of other putative σ^P^-regulated genes. Wild-type B. thuringiensis strains with the transcriptional fusion P*_sigP_*-*lacZ* (THE2549), P*_BT2718_*-*lacZ* (EBT871), P*_BT1021_*-*lacZ* (EBT873), P*_dltX_*-*lacZ* (EBT875), P*_dltC_*-*lacZ* (EBT877), or P*_BT0552_*-*lacZ* (EBT879) or B. thuringiensis *sigP-rsiP* deletion strains containing the transcriptional fusion P*_sigP_*-*lacZ* (EBT240), P*_BT2718_*-*lacZ* (EBT872), P*_BT1021_*-*lacZ* (EBT874), P*_dltX_*-*lacZ* (EBT876), P*_dltC_*-*lacZ* (EBT878), or P*_BT0552_*-*lacZ* (EBT880) were grown in the presence and absence of the inducer cefoxitin as described in Materials and Methods. Values represent the fold cefoxitin induction level where the expression of each strain grown in the presence of cefoxitin is divided by the expression of each strain grown in the absence of cefoxitin. These experiments were done in biological and technical triplicate, and standard deviation is represented by error bars. The statistical test one-way analysis of variance (ANOVA) was done using GraphPad Prism 9. *, *P* < 0.05; ***, *P* < 0.0005; ****, *P* < 0.0001.

It was previously shown that the promoters of *bla1* and *bla2* of B. anthracis were induced by the β-lactam ampicillin in a σ^P^-dependent manner in B. anthracis, B. cereus, and B. thuringiensis (20). In B. thuringiensis HD73, there are 4 annotated β-lactamases (27, 28). Our proteomic analysis demonstrated that the level of each of these 4 proteins was higher in a σ^P^-overexpressing mutant than in a *sigP* deletion strain (Table 1). To confirm that the β-lactamases of B. thuringiensis were dependent upon σ^P^, we tested each promoter for expression in the presence or absence of σ^P^. We used cefoxitin as a model inducer since it induces σ^P^ activation but *sigP* mutants are only slightly more sensitive to cefoxitin (23). We found that each promoter fusion was induced in wild type in the presence of the β-lactam cefoxitin (Fig. 1A). However, in the absence of σ^P^, expression of the β-lactamases was not induced in the presence of cefoxitin (Fig. 1A). These data show that the four annotated β-lactamases of B. thuringiensis are induced by β-lactams in σ^P^-dependent manner.

Our proteomics data suggested that expression of two putative PBPs was controlled by σ^P^ (Table 1). This includes PbpN (BT3491), which is a predicted class bPBP, and BT2717, which is homologous to DacC, a carboxypeptidase in the class C family of low-molecular-weight PBPs (5, 29, 30). We found that the expression levels of P*_dacC_-lacZ* and P*_pbpN_-lacZ* were induced by the β-lactam cefoxitin (Fig. 1B). In the absence of σ^P^, we did not observe induction of *dacC* or *pbpN* expression (Fig. 1B).

There are two additional PBPs encoded near the *sigP* loci which we did not detect in our proteomics screen but which we hypothesized may also be controlled by σ^P^ (20, 23, 24). We found that *pbpM* (*bt3487*) expression was induced by cefoxitin in a σ^P^-dependent manner like *pbpN* and *dacC*. In contrast, expression of *pbpP* was not induced by cefoxitin, nor was its expression affected by the presence or absence of σ^P^ (Fig. 1B) (24). Taken together, these data suggest that σ^P^ activity is required for expression of two class bPBP genes and a class C PBP gene.

Our proteomics analysis identified several other proteins which were more highly expressed in the *rsiP^1-60^* mutant (constitutive σ^P^ activity) than in the Δ*sigP-rsiP* mutant strain. These proteins were not predicted to bind β-lactams, nor were they predicted to have β-lactamase activity. We fused the putative promoter regions of each of these genes to *lacZ*. Of these additional genes, we found that only one was induced by cefoxitin in a σ^P^-dependent manner: *bt2718*, which encodes a putative SGNH (Ser-Gly-Asn-His) hydrolase (Fig. 1C). This suggests that most of the proteins predicted to be controlled by σ^P^ by proteomics are induced by σ^P^ in response to β-lactam antibiotics.

### Putative consensus σ^P^ promoter.

**FIG 2:**
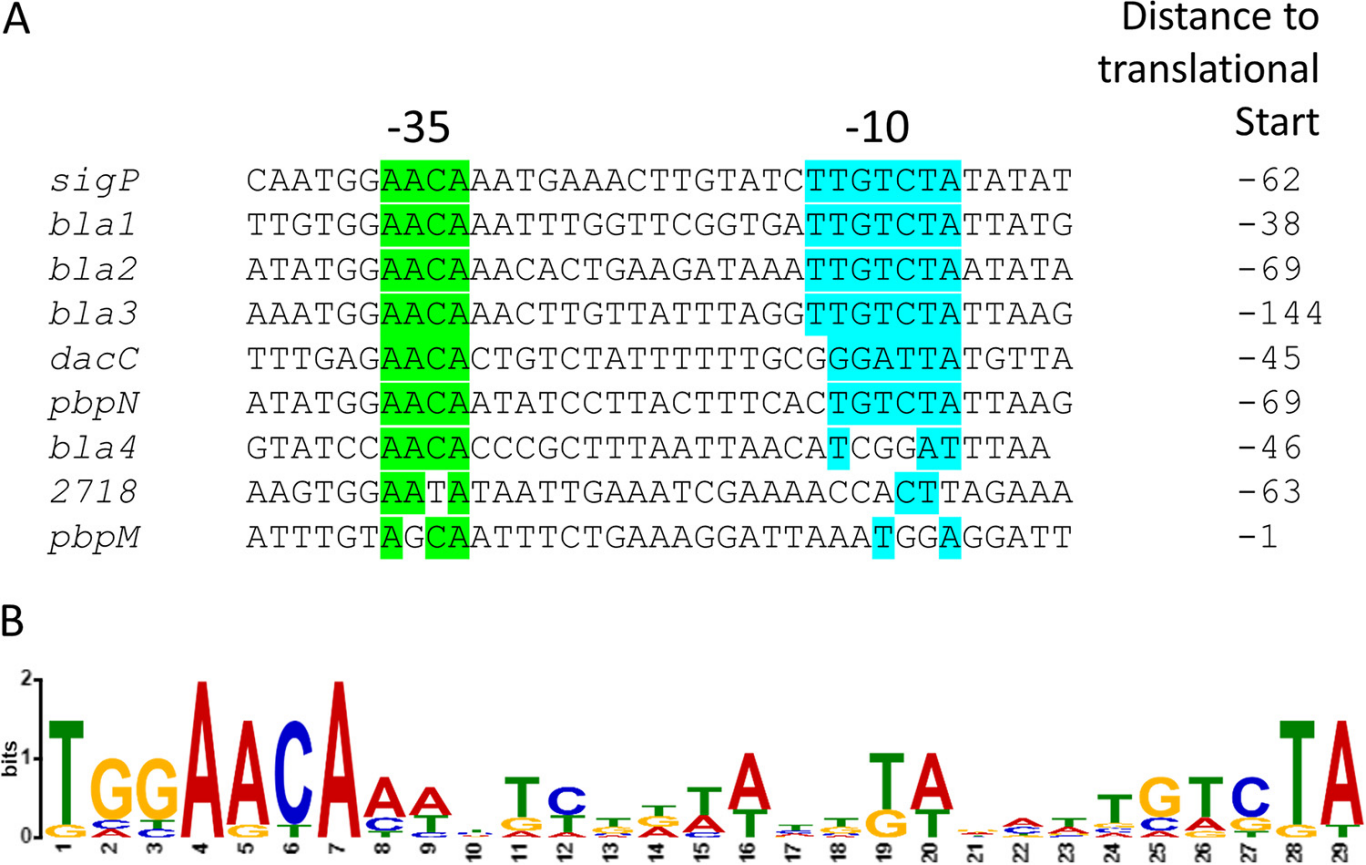
σ^P^-regulated promoters. (A) Alignment of promoter region of σ^P^-regulated genes. The promoter regions of the 9 genes confirmed to be dependent upon σ^P^ were aligned using Multiple Em for Motif Elicitation (MEME) (32). Green highlights conserved residues in the −35 region, and blue highlights conserved residues in the −10 region. (B) Consensus σ^P^ dependent promoter. The consensus sequence was generated using WebLogo (54).

Recently, σ^P^ was reclassified from ECF30 into the ECF265c2 family (31). The Koehler group used the upstream 5′ regions of the *bla1*, *bla2*, and *sigP* genes from B. anthracis to assemble a consensus (20). For these three promoters, the −35 and −10 regions were identical (20). Outside of these regions, the sequences varied in only 2 bp for each promoter (20). To generate a consensus using more diverse σ^P^ promoters, we aligned the upstream regions of the 9 genes confirmed to be dependent upon σ^P^ using Multiple Em for Motif Elicitation (MEME) (32) (Fig. 2A). From this analysis we compiled a σ^P^ consensus promoter (Fig. 2B). We found that there was strong consensus in the −35 promoter region AACA, which was similar to the previous ECF30 promoter predictions (33). Many ECF σ factors have a conserved −35 that contains AAC motif, including ECF30 and some ECF265 families (31). In contrast, we found that the −10 region had a higher degree of variation among the nine σ^P^-regulated promoters but there is a highly conserved TA. This TA is also conserved in the ECF30 −10 consensus promoter (33).

### Role of σ^P^-dependent β-lactamases in β-lactam resistance.

**FIG 3:**
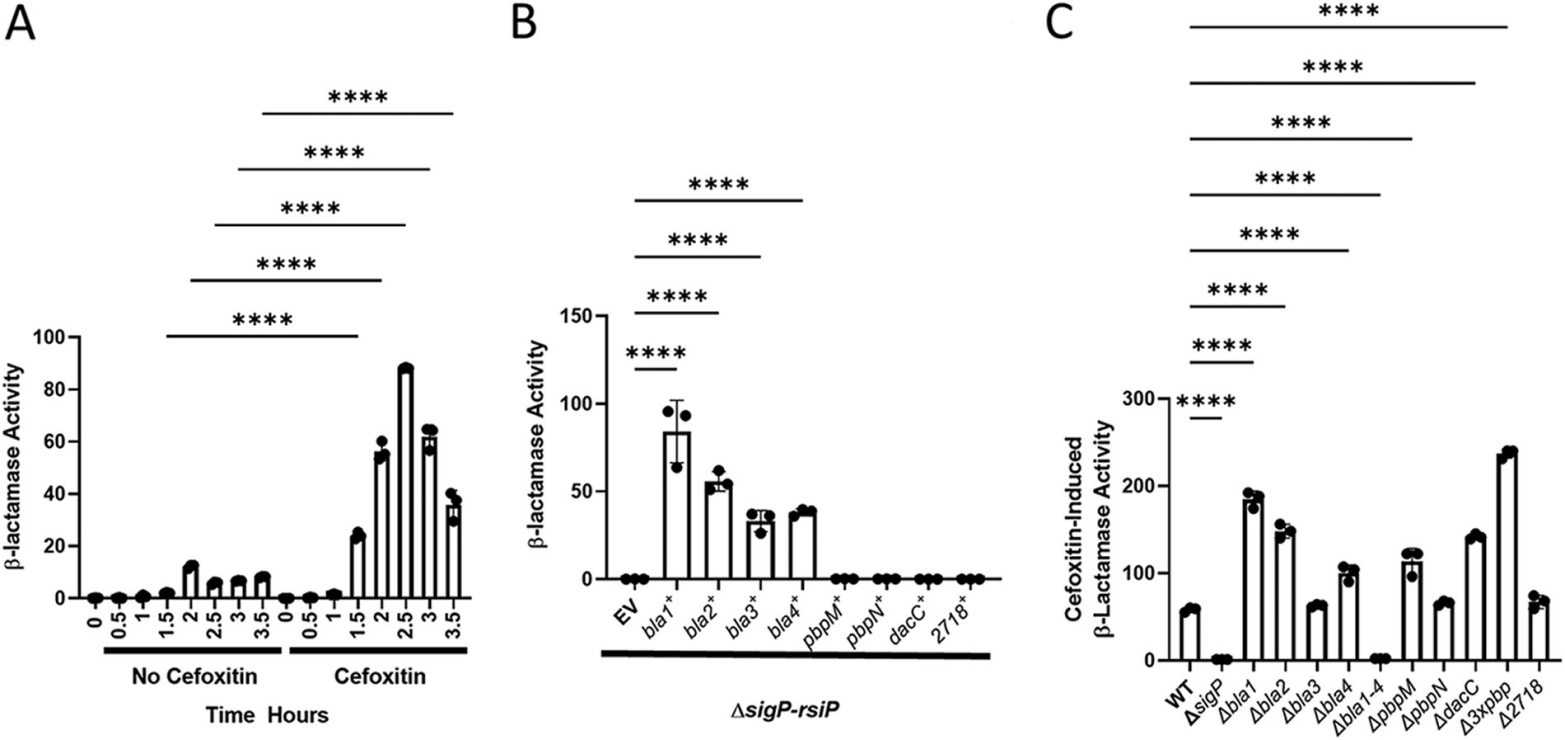
β-Lactamase activity. β-Lactamase activity was measured using the colorimetric β-lactam substrate nitrocefin. (A) β-Lactamase activity increases in the presence of cefoxitin over time. A 1:100 subculture of the wild-type strain (THE2549) was grown to an OD of 0.6 and left untreated or treated with the inducer cefoxitin (1 μg/mL). Aliquots were removed and cells were exposed to nitrocefin. Degradation of nitrocefin was detected spectrophotometrically at a wavelength of 490 nm. Values for β-lactamase activity were normalized to cell density. (B) In the absence of the σ^P^ regulon, expression of some σ^P^-regulated genes can increase β-lactamase activity. Each σ^P^-regulated gene was integrated into the chromosomal integrating conjugative element (ICE) location behind an IPTG-inducible promoter. Each strain contained a deletion of *sigP-rsiP*. A 1:100 subculture of each strain (empty vector CDE3243 [EV], *bla1^+^* CDE3245, *bla2^+^* CDE3428, *bla3^+^* CDE3429, *bla4^+^* CDE3427, *pbpM^+^* CDE3244, *pbpN^+^* CDE3212, *dacC^+^* CDE3247, and *bt72718^+^* EBT1017) was grown with the inducer IPTG (1 mM) to an OD of 1. Degradation of nitrocefin was detected spectrophotometrically at a wavelength of 490 nm. Values for β-lactamase activity were normalized to cell density. (C) Deletion of some σ^P^-regulated genes can affect β-lactamase activity. Deletions of each σ^P^-regulated gene and combinations of multiple deletions were tested was for β-lactamase activity. A 1:100 subculture of each strain (wild-type THE2549, Δ*sigP-rsiP* EBT240, Δ*bla1* EBT215, Δ*bla2* EBT652, Δ*bla3* EBT826, Δ*bla4* EBT653, Δ*bla1-4* EBT656, Δ*pbpM* EBT161, Δ*pbpN* EBT239, Δ*dacC* EBT260, Δ3×*pbp* [Δ*pbpM* Δ*pbpN* Δ*dacC*] EBT633, and Δ*2718* EBT636) was grown to an OD of 0.6 and then treated with the inducer cefoxitin (1 μg/mL) for 3 h. Degradation of nitrocefin was detected spectrophotometrically at a wavelength of 490 nm. Values for β-lactamase activity were normalized to cell density. The statistical test ANOVA was done using GraphPad Prism 9. ****, *P* < 0.0001.

To determine which β-lactamases contribute to the response of B. thuringiensis to β-lactams, we first measured the level and timing of β-lactamase activity in the wild type in the presence and absence of cefoxitin. We tested supernatants for β-lactamase activity using nitrocefin, a cephalosporin which when cleaved by a β-lactamase changes from yellow to red which can be quantitated spectrophotometrically at an absorbance wavelength of 490 nm (34). Overnight cultures of wild-type B. thuringiensis were washed and diluted in LB or LB plus cefoxitin (1 μg/mL). Samples were removed every 30 min and culture supernatants were tested for β-lactamase activity using the nitrocefin assay. In the absence of the σ^P^ inducer cefoxitin, we observed very little β-lactamase activity (Fig. 3B). We found that in the wild-type strain, measurable β-lactamase activity began at 1.5 h after cefoxitin induction and peaked at 2.5 h (Fig. 3A). This suggests that β-lactamase production in B. thuringiensis is induced by β-lactams and this induction requires σ^P^.

To determine which of the genes encode functional β-lactamases, we placed each σ^P^-dependent gene under the control of an isopropyl-β-d-thiogalactopyranoside (IPTG)-inducible promoter on a vector which was integrated into the *ICEbs1* element of a B. subtilis donor strain (35). Each vector was conjugated from a B. subtilis conjugation donor into the B. thuringiensis Δ*sigP-rsiP* mutant. The resulting transconjugants harbor the gene of interest under IPTG control at the conserved *ICEbs1* attachment site in the B. thuringiensis genome (35). We grew strains to mid-log phase in the presence of 1 mM IPTG to induce expression of the genes of interest and then tested for β-lactamase activity in a nitrocefin assay. We found that expression of each individual *bla* gene exhibited substantial β-lactamase activity (Fig. 3B). In contrast, strains expressing the other σ^P^-regulated genes *pbpM*, *pbpN*, *dacC*, and *bt2718* showed no increase in β-lactamase activity compared to the empty-vector control strain. This suggests that all four *bla* genes encode functional β-lactamases.

To determine the relative contribution of each *bla* gene to the overall β-lactamase production of B. thuringiensis, we constructed single in-frame deletion mutants of each of the four β-lactamase genes and a quadruple mutant (Δ*bla1-4*) with in-frame deletions of all four σ^P^ regulated *bla* genes. Each of these 5 mutant strains plus the wild type and a Δ*sigP-rsiP* mutant strain were grown for 3 h in the presence of cefoxitin. Culture supernatants were then tested for β-lactamase activity using the nitrocefin cleavage assay. All single *bla* mutants showed β-lactamase activity similar to that of the wild type (Fig. 3B). The quadruple *bla* mutant showed no detectable β-lactamase activity, similar to the *sigP* mutant (Fig. 3B). Taken together, these data suggest that multiple β-lactamases can cleave β-lactams like nitrocefin and that no single *bla* gene was responsible for all of the β-lactamase activity induced by σ^P^. It also suggests that we have identified all of the σ^P^-dependent β-lactamases in B. thuringiensis.

### Sufficiency of σ^P^-regulated genes for β-lactam resistance.

The σ^P^ regulon induces 50-fold or greater resistance to the β-lactams ampicillin, methicillin, cephalexin, and cephalothin (23). We choose to focus on these antibiotics because the σ^P^ regulon has the greatest effect on resistance to these β-lactams, as previously described (23). To test which of the σ^P^-regulated genes was sufficient to provide β-lactam resistance, we determined the MICs of a panel of strains expressing each gene of interest under the control of an IPTG-inducible promoter in a Δ*sigP-rsiP* mutant. We compared the MIC of each gene of interest to that of the empty vector control and determined the fold difference in MIC. We defined high-level resistance as a >100-fold increase, moderate-level resistance as a 10- to 100-fold increase, and low-level resistance as a 2- to 10-fold increase over the Δ*sigP-rsiP* mutant containing the vector control.

**FIG 4:**
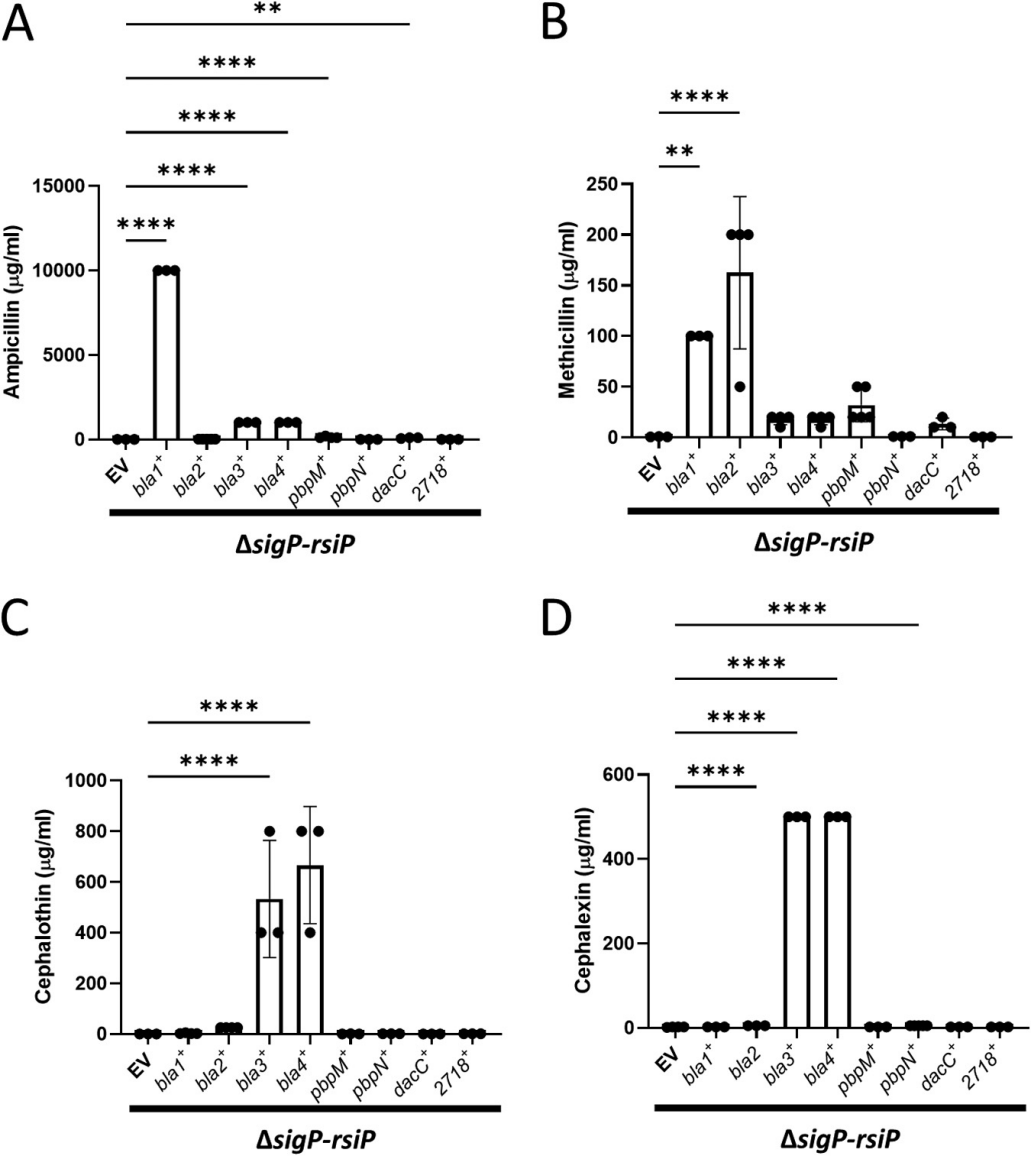
Antibiotic resistance profile of overexpression of σ^P^-regulated genes in a Δ*sigP-rsiP* deletion strain. An overnight culture of each Δ*sigP-rsiP* strain containing an overexpression plasmid (EV CDE3243, *bla1^+^* CDE3245, *bla2^+^* CDE3428, *bla3^+^* CDE3429, *bla4^+^* CDE3427, *pbpM^+^* CDE3244, *pbpN^+^* CDE3212, *dacC^+^* CDE3247, or *bt72718^+^* EBT1017) was grown in the absence of IPTG at 37°C. Each strain was washed in fresh medium and subcultured into medium containing 1 mM IPTG and various concentrations of antibiotic and incubated overnight at 37^o^C. Overexpression of some σ^P^-regulated genes was sufficient to increase ampicillin (A), methicillin (B), cephalothin (C), or cephalexin (D) resistance. The statistical test ANOVA was done using GraphPad Prism 9. **, *P* < 0.005; ****, *P* < 0.0001.

For ampicillin, we found that expression of *bla1* provided high-level resistance (Fig. 4A). Expression of *bla3* and *bla4* resulted in moderate-level resistance (Fig. 4A). Production of Bla2 had a slight effect on ampicillin resistance. These data suggest that Bla1, Bla3, and Bla4 are sufficient for conferring ampicillin resistance. We found that methicillin resistance was provided by a different array of σ^P^-regulated genes. Bla1 and Bla2 expression resulted in high-level methicillin resistance (Fig. 4B). Expression of PbpM, Bla3, Bla4 and DacC resulted in a moderate increase in methicillin resistance (Fig. 4B).

In the case of the first-generation cephalosporin β-lactams cephalothin and cephalexin, Bla3 and Bla4 expression resulted in high levels of antibiotic resistance (Fig. 4C and D). Additionally, Bla2 had a moderate effect on cephalothin resistance (50-fold) but not to the same degree as Bla3 and Bal4 (1,067-fold and 1,333-fold, respectively [Fig. 4D]). Overexpression of *bt2718* had no effect on resistance to any of the β-lactams tested. Taken together, our data present a picture of how an arsenal of σ^P^-regulated proteins allows B. thuringiensis to withstand a range of β-lactam antibiotics.

### Necessity of σ^P^-regulated genes for β-lactam resistance.

While many of the σ^P^-regulated genes are sufficient to increase β-lactam resistance when produced in the absence of σ^P^, we wanted to determine which genes were required for resistance to β-lactams. To explore necessity, we tested single, in-frame deletions of each of the σ^P^-regulated genes. Additionally, we constructed and tested a triple PBP mutant, the Δ3*×pbp* mutant, which lacked *pbpM*, *pbpN*, and *dacC*, and the Δ*bla1-4* quadruple mutant, in which we deleted *bla1*, *bla2*, *bla3*, and *bla4*. These strains were tested in MIC experiments to determine their levels of resistance to the β-lactams ampicillin, methicillin, cephalothin, and cephalexin.

**FIG 5:**
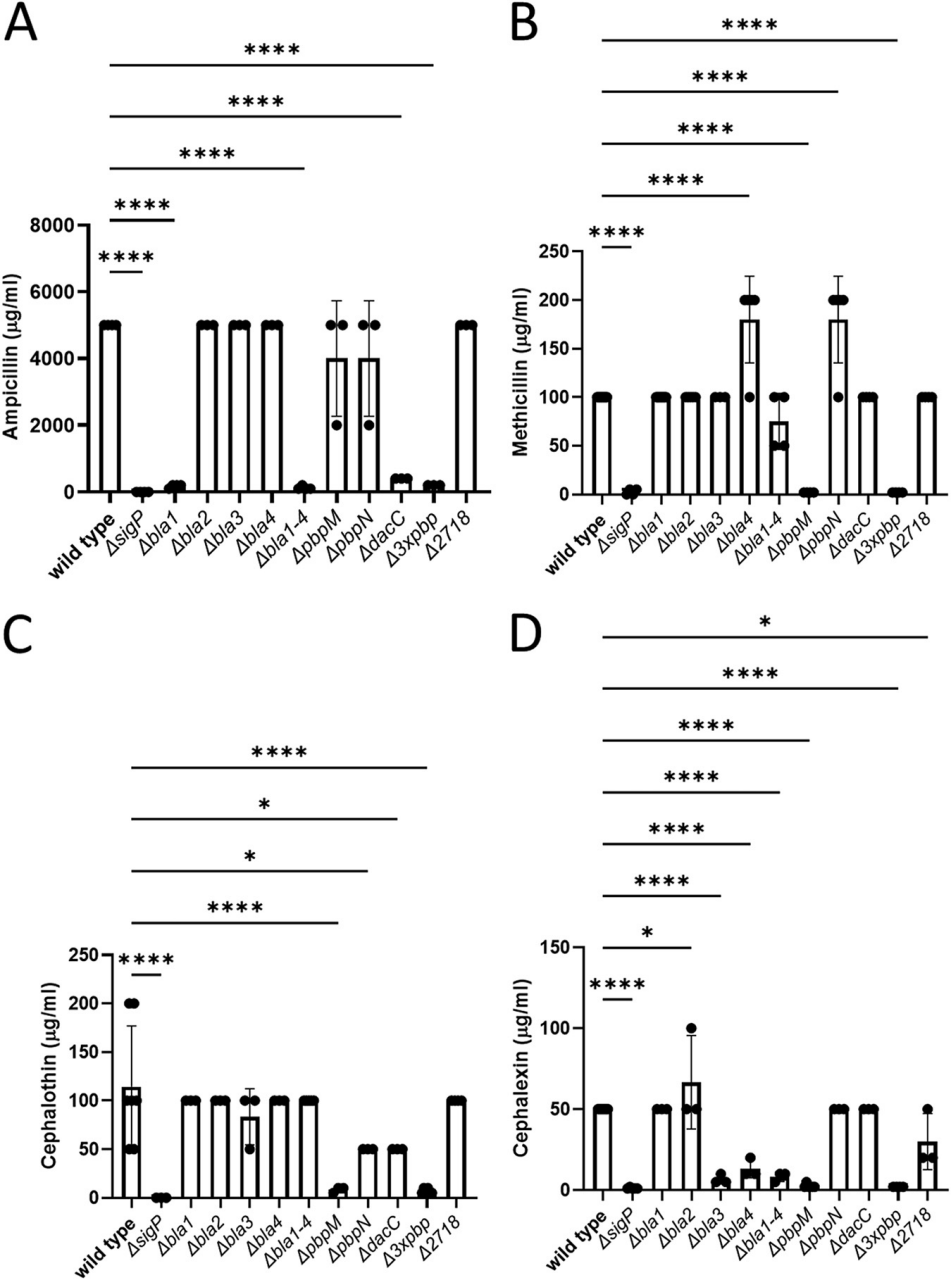
Antibiotic resistance profile of deletion of σ^P^-regulated genes. An overnight culture of each deletion strain (wild-type THE2549, Δ*sigP-rsiP* EBT240, Δ*bla1* EBT215, Δ*bla2* EBT652, Δ*bla3* EBT826, Δ*bla4* EBT653, Δ*bla1-4* EBT656, Δ*pbpM* EBT161, Δ*pbpN* EBT239, Δ*dacC* EBT260, Δ3×*pbp* [Δ*pbpM* Δ*pbpN* Δ*dacC*] EBT633, and Δ*2718* EBT636) was grown overnight at 37°C. Each strain was washed in fresh medium and subcultured into media containing various concentrations of antibiotic and incubated overnight at 37°C. Loss of some σ^P^-regulated genes decreased resistance to ampicillin (A), methicillin (B), cephalothin (C), or cephalexin (D). The statistical test one-way ANOVA was done using GraphPad Prism 9. *, *P* < 0.05; ****, *P* < 0.0001.

We found that the β-lactamase Bla1 and the PBP DacC were required for ampicillin resistance (Fig. 5A). The *bla1* mutant strain had a 28-fold-lower ampicillin MIC than did the wild type. The ampicillin MIC of the quadruple *bla* mutant was similar to that of the single *bla1* deletion strain (Fig. 5A). The ampicillin MICs of the *dacC* mutant and the triple PBP mutant were 12.5-fold and 25-fold lower than the wild type MIC, respectively (Fig. 5A). These data suggest that *bla1* and *dacC* are required for wild-type levels of ampicillin resistance and that other β-lactamases and PBPs do not significantly contribute to ampicillin resistance.

Methicillin resistance seems to be independent of the β-lactamases, since deletion of any one or all the β-lactamase genes did not have an effect on resistance (Fig. 5B). In contrast, deletion of *pbpM* resulted in a 50-fold reduction in methicillin resistance compared to that of the wild type. The triple PBP mutant had a phenotype similar to that of the *pbpM* single mutant (Fig. 5B), suggesting that only *pbpM* is necessary for full methicillin resistance.

Like resistance to methicillin, resistance to cephalothin and cephalexin relied heavily on *pbpM*. The *pbpM* mutant was 14-fold and 20-fold less resistant to cephalothin and cephalexin, respectively (Fig. 5C and D). Similarly, the triple PBP mutant was 17-fold and 25-fold less resistant to these antibiotics (Fig. 5C and D). These data suggest that the only PBP which is required for resistance to these second-generation cephalosporins is PbpM.

Similar to the case with methicillin, cephalothin resistance was not altered by any single *bla* mutant or the quadruple *bla* deletion (Fig. 5C). In contrast, cephalexin resistance is likely due to a combination of β-lactamases and PBPs. The *bla3* and *bla4* mutants were slightly each more sensitive to cephalexin than the wild type (7.5-fold and 3.8-fold, respectively [Fig. 5D]). The quadruple *bla* mutant exhibited the same phenotype as the *bla3* and *bla4* single mutants (Fig. 5D). Deletion of *bt2718* had no effect on resistance to any of the β-lactams tested.

Taken together, our data suggest that the σ^P^ regulon controls a variety of genes that are involved in resistance to β-lactam-mediated killing. Activation of σ^P^ increases expression of multiple β-lactam resistance genes, and no single gene is responsible for resistance to all β-lactams. Thus, activation of σ^P^ controls resistance to a wide variety of β-lactams because it induces so many different β-lactam resistance genes.

## DISCUSSION

Our work shows that σ^P^ activity controls expression of multiple genes which protect B. thuringiensis from β-lactam-mediated killing. σ^P^ is required for transcription of genes encoding two broad classes of enzymes: β-lactamases, which degrade β-lactam antibiotics, and penicillin-binding proteins, which are the targets of β-lactams. By overexpressing each σ^P^-regulated gene independently of other σ^P^-regulated genes, we were able to determine the potential resistance that each component could provide. We also tested the relative contribution of each σ^P^-regulated component to β-lactam resistance by deleting each component individually. Using these data, we determined which proteins are necessary for B. thuringiensis survival in the presence of different β-lactam antibiotics.

Other signaling systems that control β-lactamase gene expression in response to β-lactams have been described for Gram-positive bacteria, like Staphylococcus aureus and Bacillus licheniformis. However, in this example, expression of single a β-lactamase gene is controlled by the regulatory system (36). BlaRI controls expression of *blaZ*, which encodes a β-lactamase (36). BlaR (MecR) contains an extracellular transpeptidase-like domain that senses β-lactams (37). Binding of the β-lactam ring causes a conformational change in BlaR which activates the cytoplasmic protease domain (36). The protease domain cleaves the repressor of the β-lactamase operon, BlaI, thus activating transcription of β-lactamase and increasing resistance to β-lactams (36).

Previous work from our lab showed that σ^P^ activity is controlled by PbpP, which also directly binds to β-lactams (24). However, unlike BlaRI, σ^P^ controls expression of four different β-lactamases, two PBPs, and one carboxypeptidase. It is unclear why B. thuringiensis and related organisms encode so many β-lactam resistance mechanisms and why they utilize the same signal transduction system to control all of them. One possible explanation is that in the natural environment, B. thuringiensis and related organisms encounter a range of β-lactams and cephalosporins. Thus, they utilize a single system to sense and induce resistance to a wide variety of β-lactam antibiotics.

### β-Lactamases.

β-Lactamases hydrolyze the β-lactam ring. The ability to hydrolyze a β-lactam ring can be measured using the substrate nitrocefin. We found that all 4 annotated σ^P^-regulated β-lactamases can cleave nitrocefin. When overexpressed individually, each of these β-lactamases was sufficient to increase resistance to β-lactams, although there were clear differences in the preferred substrates. Bla1 increased resistance to ampicillin and methicillin but not cephalexin and cephalothin. Bla2 protected only against methicillin. Bla3 and Bla4 expression resulted in high levels of resistance to cephalexin and cephalothin and moderate levels of resistance to ampicillin and methicillin. This suggests that these β-lactamases have different specificities for classes of β-lactam antibiotics.

### Testing of individual deletions of the 4 *bla* genes for their necessity in β-lactam resistance.

Deletion of either *bla3* or *bla4* resulted in decreases in cephalexin resistance. Overexpression of Bla3 and Bla4 provided increased resistance to cephalexin. Thus, these β-lactamases were necessary and sufficient to protect against cephalexin assault. Bla3 and Bla4 overexpression were also sufficient to provide resistance to cephalothin. However, deletions in one or all *bla* genes had no effect on cephalothin resistance. These data suggest that while Bla3 and Bla4 can provide resistance, other mechanisms could protect against cephalothin in their absence. Bla3 and Bla4 are class A β-lactamases. Based on sequence alignment, Bla3 and Bla4 are 75% identical on the protein level. Given the similarity in sequence, it is not surprising that Bla3 and Bla4 have similar activities against the 4 different β-lactams tested in this study. It is likely that they provide redundant protection against β-lactams.

Like Bla3 and Bla4, Bla1 is a class A β-lactamase. However, Bla1 is less similar to Bla3 and Bla 4 (53% and 57% protein identities, respectively). Overexpression of Bla1 substantially increased resistance to ampicillin and methicillin. However, we found that *bla1* was only required for full ampicillin resistance. This suggests that a redundant methicillin resistance mechanism is active in the absence of *bla1*.

Based on sequence analysis, Bla2 is a class B metallo-β-lactamase (13). Bla2 can provide resistance to methicillin when overexpressed. However, deletion of *bla2* does not significantly lower resistance to any of the β-lactams tested, including methicillin. Given its substrate specificity, it is likely in the 3a functional group of β-lactamases (13). Our data suggest that multiple redundant σ^P^-regulated mechanisms provide resistance to methicillin killing.

One issue that may complicate the interpretation of the necessity experiments is that disruption of resistance mechanisms likely alters the response of the σ^P^ regulatory system. For example, removing a β-lactamase could result in less degradation of the β-lactam, which would allow for increased activation of σ^P^ and thus increased expression of other β-lactamases and PBPs. Thus, the effect of deletion of a β-lactamase gene could be masked by a corresponding increase in σ^P^ activation. In fact, we previously observed that deletion of *bla1* resulted in increased activation of σ^P^ in response to ampicillin (23). While this could clearly complicate some interpretations, our data show distinct examples of β-lactamases that are unambiguously required for resistance to β-lactams.

### Modified β-lactam target transpeptidases (production of alternative PBPs).

Bacteria can also evade antibiotics by modifying the bacterial target of an antibiotic. With β-lactams, this occurs by production of a PBP that has decreased affinity to the β-lactam in question (11). This can occur by either mutation or increased expression of an alternative PBP which is less sensitive to the β-lactam (11). We found that two of the σ^P^-regulated PBPs were involved in resistance to the β-lactams we tested. Deletion of *pbpM* increased sensitivity to methicillin, cephalexin, and cephalothin. PbpM is a class B PBP and is likely capable of transpeptidation. Thus, we hypothesize that PbpM has a decreased ability to bind methicillin, cephalexin, and cephalothin than normal “housekeeping” PBPs and thus provides increased resistance to these antibiotics. We have found that an additional class B PBP (PbpN) is also σ^P^ regulated. Deletion or overexpression of the *pbpN* gene had no effect on resistance to ampicillin, methicillin, cephalothin, or cephalexin. It is possible that PbpN can provide resistance to β-lactams which we have not yet tested. By producing antibiotic-resistant PBPs, the σ^P^ system enables the bacteria to continue performing necessary transpeptidase functions.

### Other mechanisms of resistance to β-lactams.

Although DacC is predicted to bind penicillin, it is likely not a transpeptidase. DacC is predicted to be a class C PBP and is likely a carboxypeptidase responsible for removing d-Ala from the pentapeptide side chain of peptidoglycan based on sequence homology. Deletion of *dacC* decreased resistance to ampicillin. There are other examples where loss of a peptidoglycan carboxypeptidase leads to increased β-lactam resistance (38–40). In some of these cases, it appears to cause a shift to 3-3 cross-linking of the peptide side chains of peptidoglycan, which is carried out by Ldts. Ldts are structurally distinct forms of PBPs, are resistant to most β-lactams, and cross-link a tetramer side chain to the peptidoglycan (38). DacC likely increases the pool of Ldt substrates, thus increasing β-lactam resistance. B. anthracis and B. thuringiensis encode putative Ldts which are likely resistant to β-lactams; however, they have not been investigated (41).

The BT2718 open reading frame is predicted to encode an SGNH/GDSL hydrolase. In our studies, overexpression of BT2718 did not result in nitrocefin hydrolysis, nor did it affect resistance to ampicillin, methicillin, cephalexin, or cephalothin. Some esterases cannot hydrolyze the β-lactam ring but are able to deacetylate some cephalosporins (42, 43). For instance, Burkholderia gladioli SGNH/GDSL hydrolase EstB is unable to hydrolyze the β-lactam ring of nitrocefin but can deacetylate cephalosporin C, cephalothin, and 7-aminocephalosporanic acid (7-ACA) (44, 45). Hydrolyzing the acetyl group from these cephalosporin derivatives modifies the R2 side chain, which generates modified β-lactams (44). These modified β-lactams lose the ability to inhibit PBP activity. While we did not find a role for BT2718 in resistance to β-lactams, it is possible that BT2718 has different substrate specificity and that, if given the appropriate substrate, BT2718 could modify that β-lactam.

## MATERIALS AND METHODS

### Media and growth conditions.

All B. thuringiensis strains used in this study are isogenic derivatives of AW43, a derivative of Bacillus thuringiensis subsp. *kurstaki* strain HD73 (28). All strains and genotypes can be found in Table 2. All B. thuringiensis strains were grown in or on LB media at 30°C unless otherwise specified. Cultures of B. thuringiensis were grown with agitation in a roller drum. Strains containing episomal plasmids were grown in LB containing chloramphenicol (Cam; 10 μg/mL) or erythromycin (Erm, 10 μg/mL). Escherichia coli strains were grown at 37°C using LB-ampicillin (Amp; 100 μg/mL) or LB-Cam (10 μg/mL) medium. To screen for threonine auxotrophy, B. thuringiensis strains were patched on minimal medium plates without or with threonine (50 μg/mL) (46, 47). The β-galactosidase chromogenic indicator 5-bromo-4-chloro-3-indolyl β-d-galactopyranoside (X-Gal) was used at a concentration of 100 μg/mL.

**TABLE 2:** Strains

Strain	Description	Reference
*B. thuringiensis*		
AW43	*B. thuringiensis* serovar kurstaki HD73 cured of both pAW63 and pHT73; Nal^r^	28
THE2549	AW43 *thrC*::P*_sigP_-lacZ*	23
EBT240	AW43 *thrC*::P*_sigP_-lacZ* Δ*sigP-rsiP*	23
THE2548	AW43 *thrC*::P*_pbpM_-lacZ*	This study
EBT316	AW43 *thrC*::P*_pbpM_-lacZ* Δ*sigP-rsiP*	This study
EBT234	AW43 *thrC*::P*_pbpP_-lacZ*	This study
EBT477	AW43 *thrC*::P*_pbpP_-lacZ* Δ*sigP-rsiP*	This study
THE2547	AW43 *thrC*::P*_pbpN_-lacZ*	This study
EBT315	AW43 *thrC*::P*_pbpN_-lacZ* Δ*sigP-rsiP*	This study
EBT221	AW43 *thrC*::P*_dacC_-lacZ*	This study
EBT480	AW43 *thrC*::P*_dacC_ -lacZ* Δ*sigP-rsiP*	This study
EBT228	AW43 *thrC*::P*_bla1_-lacZ*	This study
EBT479	AW43 *thrC*::P*_bla1_-lacZ* Δ*sigP-rsiP*	This study
EBT322	AW43 *thrC*::P*_bla2_-lacZ*	This study
EBT324	AW43 *thrC*::P*_bla2_-lacZ* Δ*sigP-rsiP*	This study
EBT208	AW43 *thrC*::P*_bla3_-lacZ*	This study
EBT210	AW43 *thrC*::P*_bla3_-lacZ* Δ*sigP-rsiP*	This study
EBT869	AW43 *thrC*::P*_bla4_-lacZ*	This study
EBT870	AW43 *thrC*::P*_bla4_-lacZ* Δ*sigP-rsiP*	This study
EBT871	AW43 *thrC*::P*_bt2718_-lacZ*	This study
EBT872	AW43 *thrC*::P*_bt2718_-lacZ* Δ*sigP-rsiP*	This study
EBT873	AW43 *thrC*::P*_bt1021_-lacZ*	This study
EBT874	AW43 *thrC*::P*_bt1021_-lacZ* Δ*sigP-rsiP*	This study
EBT875	AW43 *thrC*::P*_dltX_-lacZ*	This study
EBT876	AW43 *thrC*::P*_dltX_-lacZ* Δ*sigP-rsiP*	This study
EBT877	AW43 *thrC*::P*_dltC_-lacZ*	This study
EBT878	AW43 *thrC*::P*_dltC_-lacZ* Δ*sigP-rsiP*	This study
EBT879	AW43 *thrC*::P*_bt0552_-lacZ*	This study
EBT880	AW43 *thrC*::P*_bt0552_-lacZ* Δ*sigP-rsiP*	This study
CE3243	AW43 *thrC*::P*_sigP_-lacZ* Δ*sigP-rsiP* ICE::pCE697 (P_IPTG_)	This study
CE3245	AW43 *thrC*::P*_sigP_-lacZ* Δ*sigP-rsiP* ICE::pCE727 (P_IPTG_-*bla1)*	This study
CE3428	AW43 *thrC*::P*_sigP_-lacZ* Δ*sigP-rsiP* ICE::pCE809 (P_IPTG_-*bla2*)	This study
CE3429	AW43 *thrC*::P*_sigP_-lacZ* Δ*sigP-rsiP* ICE::pCE810 (P_IPTG_-*bla3*)	This study
CE3427	AW43 *thrC*::P*_sigP_-lacZ* Δ*sigP-rsiP* ICE::pCE808 (P_IPTG_-*bla4*)	This study
CE3244	AW43 *thrC*::P*_sigP_-lacZ* Δ*sigP-rsiP* ICE::pCE699 (P_IPTG_-*pbpM*)	This study
CE3212	AW43 *thrC*::P*_sigP_-lacZ* Δ*sigP-rsiP* ICE::pCE708 (P_IPTG_-*pbpN*)	This study
CE3247	AW43 *thrC*::P*_sigP_-lacZ* Δ*sigP-rsiP* ICE::pCE729 (P_IPTG_-*dacC*)	This study
EBT1017	AW43 *thrC*::P*_sigP_-lacZ* Δ*sigP-rsiP* ICE::pCE821 (P_IPTG_-*bt2718*)	This study
EBT215	AW43 *thrC*::P*_sigP_-lacZ* Δ*bla1*	23
EBT652	AW43 *thrC*::P*_sigP_-lacZ* Δ*bla2*	This study
EBT648	AW43 *thrC*::P*_sigP_-lacZ* Δ*bla3*	This study
EBT653	AW43 *thrC*::P*_sigP_-lacZ* Δ*bla4*	This study
EBT656	AW43 *thrC*::P*_sigP_-lacZ* Δ*bla3* Δ*bla4* Δ*bla2* Δ*bla1*	This study
EBT161	AW43 *thrC*::P*_sigP_-lacZ* Δ*pbpM*	This study
EBT239	AW43 *thrC*::P*_sigP_-lacZ* Δ*pbpN*	This study
EBT260	AW43 *thrC*::P*_sigP_-lacZ* Δ*dacC*	This study
EBT633	AW43 *thrC*::P*_sigP_-lacZ* Δ*pbpM* Δ*pbpN* Δ*dacC*	This study
EBT636	AW43 *thrC*::P*_sigP_-lacZ* Δ*bt2718*	This study
		
*E. coli*		
OmniMax 2-T1R	F′ [*proAB*^+^ *lacI*^q^ *lacZ*ΔM15 Tn*10*(Tet^r^) Δ(*ccdAB*)] *mcrA* Δ(*mrr-hsdRMS-mcrBC*) φ80(*lacZ*)ΔM15 Δ(*lacZYA-argF*)*U169* *endA1 recA1 supE44 thi-1 gyrA96 relA1 tonA panD*	

### Proteomics sample preparation.

Bacterial pellets were lysed in 8 M urea. The protein concentration of each sample was measured using a Bradford assay (Bio-Rad) and equal protein amounts, each containing 250 μg total protein, were used for further analysis. Proteins in each sample were reduced by adding 5 mM (final concentration) dithiothreitol and samples were incubated for 30 min at 55°C. Proteins were then alkylated by the addition of 10 mM (final concentration) chloroacetamide for 15 min at room temperature in the dark. Each sample was then diluted with 20 mM HEPES (pH 8.0) to a urea concentration of 4 M, and the proteins were digested with 2.5 μg LysC (Wako) (1/100, wt/wt) for 4 h under agitation at 37°C. The samples were then further diluted to a urea concentration of 2 M with 20 mM HEPES (pH 8.0) and digested with 2.5 μg trypsin (Promega) (1/200, wt/wt) overnight at 37°C. Samples were acidified to 1% trifluoroacetic acid (TFA) and desalted on reverse-phase C_18_ OMIX tips (Pierce), all according to the manufacturer’s specifications. Purified peptides were then stored at −80°C until liquid chromatography-tandem mass spectrometry (LC-MS/MS) analysis.

### LC-MS/MS and data analysis.

Purified peptides were redissolved in 15 μL loading solvent A (0.1% TFA plus 3% acetonitrile [ACN] in water). Of the resuspended peptide solution, 2 μg was injected for LC-MS/MS analysis on an RSLCnano system connected to a an Orbitrap Fusion Lumos Tribrid mass spectrometer (Thermo Scientific). Trapping was performed at 10 μL/min for 4 min in loading solvent A on a 25-mm C_18_ trapping column (New Objective). Peptides were eluted by a nonlinear increase from 2% to 56% solvent B (0.1% formic acid in water/acetonitrile [2:8, vol/vol]) over 160 min at a constant flow rate of 500 nL/min. The 200-cm column temperature was kept at a constant 37°C in a column oven.

Data analysis was performed with MaxQuant (version 1.6.10.43) using the Andromeda search engine with default search settings, including a false-discovery rate (FDR) set at 1% on both the peptide and protein levels. Spectra were compared against B. thuringiensis serovar kurstaki (NCBI taxonomy identifier [ID] 1279365) protein database containing 6,032 sequences. For both searches, a mass tolerance for precursor ions was set at 4.5 ppm, with a mass tolerance for fragment ions at 20 ppm and 0.5 Da. A maximum of 2 missed cleavages was set for both shotgun searches. Carbamidomethylation of cysteine residues was set as a fixed modification, while variable modifications were set to oxidation of methionine and acetylation of protein N termini. Only proteins with at least one unique or razor peptide were retained in both shotgun searches.

Further data analysis was performed with the Perseus software (version 1.6.14.0) after loading the protein groups sites table generated from MaxQuant. Reverse database hits were removed, as well as potential contaminants and IDs only identified by sites. Remaining hits were considered to be identifications. Intensities were log_2_ transformed and normalized for each sample by subtracting the median LFQ (label free quantitation) intensity. Replicate samples were grouped, and sites with fewer than three valid values in at least one group were removed, deeming a hit to be quantifiable. Missing values were imputed from a normal distribution around the detection limit. To reveal intensities that were significant, samples were grouped based on mutation (Δ*sigP-rsiP* versus *rsiP^1-80^*) and *t* tests (FDR = 0.05 and S0 = 1) were performed to compare the intensities of the sites quantified under each condition. From the *t* tests, identifications with *P* values of <0.01 (−log *P* values > 2), and fold change of >1 were considered significant.

### Strain and plasmid construction.

All plasmids are listed in Table 3 and expanded in Table S1 in the supplemental material. Plasmids were constructed by isothermal assembly (48). Regions of plasmids constructed using PCR were verified by DNA sequencing. The oligonucleotide primers used in this work were synthesized by Integrated DNA Technologies (Coralville, IA) and are listed in Table S2. All plasmids were propagated using the E. coli cloning host OmniMax 2-T1R before being transformed into a B. thuringiensis recipient strain.

**TABLE 3:** Plasmids

Plasmid	Relevant features	Primer pair(s)	Reference
pTHE950	pE194ts, *'thrC lacZ thrB'*		23
pTHE954	pE194ts, *'thrC* P*_pbpM_-lacZ thrB'*	2931-2932	This study
pTHE955	pE194ts, *'thrC* P*_pbpP_-lacZ thrB'*	2933-2934	This study
pTHE951	pE194ts, *'thrC* P*_pbpN_-lacZ thrB'*	2926-2927	This study
pBT7	pE194ts, *'thrC* P*_dacC_-lacZ thrB'*	2941-2942	This study
pBT9	pE194ts, *'thrC* P*_bla1_-lacZ thrB'*	2935-2936	This study
pBT8	pE194ts, *'thrC* P*_bla2_-lacZ thrB'*	2973-2974	This study
pTHE956	pE194ts, *'thrC* P*_bla3_-lacZ thrB'*	2943-2944	This study
pTHE957	pE194ts, *'thrC* P*_bla4_-lacZ thrB'*	2975-2976	This study
pTHE958	pE194ts, *'thrC* P*_bt2718_-lacZ thrB'*	2977-2978	This study
pTHE959	pE194ts, *'thrC* P*_bt1021_-lacZ thrB'*	2979-2980	This study
pCE801	pE194ts, *'thrC* P*_dltX_-lacZ thrB'*	5028-5029	This study
pCE802	pE194ts, *'thrC* P*_dltC_-lacZ thrB'*	5030-5031	This study
pCE803	pE194ts, *'thrC* P*_bt0552_-lacZ thrB'*	5032-5033	This study
pCE697	ICE*Bs1*::P*_IPTG_ amp cat*		24
pCE727	ICE*Bs1*::P*_IPTG_-bla1 amp cat*		This study
pCE809	ICE*Bs1*::P*_IPTG_-bla2 amp cat*		This study
pCE810	ICE*Bs1*::P*_IPTG_-bla3 amp cat*		This study
pCE808	ICE*Bs1*::P*_IPTG_-bla4 amp cat*		This study
pCE699	ICE*Bs1*::P*_IPTG_-pbpM amp cat*		This study
pCE708	ICE*Bs1*::P*_IPTG_-pbpN amp cat*		This study
pCE729	ICE*Bs1*::P*_IPTG_-dacC amp cat*		This study
pCE821	ICE*Bs1*::P*_IPTG_-bt2718 amp cat*		This study
pMAD	ori-pE194ts		49
pBT1	ori-pE194ts, Δ*pbpM*	3828-3829, 3830-3831	This study
pBT3	ori-pE194ts, Δ*pbpN*	3820-3821, 3822-3823	This study
pBT14	ori-pE194ts, Δ*dacC*	2948-2949, 2950-2951	This study
pCE792	ori-pE194ts, Δ*bla2*	2981-2982, 2983-2984	This study
pCE826	ori-pE194ts, Δ*bla3*	5133-5134, 5135-5136	This study
pCE793	ori-pE194ts, Δ*bla4*	2985-2986, 2987-2988	This study
pCE820	ori-pE194ts, Δ*bt2718*	5105-5106, 5107-5108	This study
pEBT4	ori-pE194ts, Δ*bla1* (Δ*blaP*)		23

10.1128/msphere.00967-21.1 TABLE S1 Plasmids used in this study Table S1, PDF file, 0.1 MB. Copyright © 2022 Ho et al. 2022 Ho et al. https://creativecommons.org/licenses/by/4.0/ This content is distributed under the terms of the Creative Commons Attribution 4.0 International license.

10.1128/msphere.00967-21.2 TABLE S2 Primers used in this study Table S2, PDF file, 0.1 MB. Copyright © 2022 Ho et al. 2022 Ho et al. https://creativecommons.org/licenses/by/4.0/ This content is distributed under the terms of the Creative Commons Attribution 4.0 International license.

### B. thuringiensis DNA transformation.

Plasmids were introduced into B. thuringiensis by electroporation as previously described (23). Briefly, recipient cell cultures inoculated from freshly isolated colonies were grown to late log phase at 37°C. For each transformation, cells (4.5 mL) were pelleted by centrifugation (9,000 × *g*) and washed twice in sterile water. After careful removal of all residual water, 100 μL sterile 40% polyethylene glycol (PEG) warmed to 42°C was used to gently resuspended cells. Approximately 0.2 to 1 μg DNA was mixed with cells and transferred to a 0.4-cm-gap electroporation cuvette (Bio-Rad). Cells were exposed to 2.5 kV for 4 to 6 ms. LB was immediately added and cells were incubated at room temperature for 1 h prior to plating on selective media.

### Construction of deletion mutant strains in B. thuringiensis.

We generated unmarked deletion mutations as previously described (23). To construct deletion mutants, we cloned 1 kb DNA upstream and 1 kb downstream of the site of desired deletion using primers listed in Table S2 onto the temperature-sensitive pMAD plasmid (erythromycin resistant) between the BglII and EcoRI sites (49). Deletion mutant plasmids were integrated into an appropriate recipient strain at a low temperature and then shifted to a high temperature to allow recircularization of the plasmid. The plasmid-containing strains were streaked several times at a low temperature until the plasmid was lost by segregation. Strains lacking the plasmid were screened for the deletion by PCR. All deletion mutant plasmids are listed in Table 3.

### Construction of deletions or promoter-*lacZ* fusions in B. thuringiensis.

We generated strains containing a promoter fused to the *lacZ* reporter integrated into the chromosome using our previously described method (23). Briefly, we cloned the regions upstream of each open reading frame onto the pTHE950 plasmid (XhoI and NotI digested). Each promoter fusion strain was integrated into the *thrC* operon as previously described. All promoter fusion plasmids are listed in Table 3, and all primers used to generate the plasmids are shown in Table S2.

### Construction of expression vectors in B. thuringiensis.

IPTG-inducible constructs utilized were constructed by cloning into the Sal and NheI sites of pCE697 (24). DNA was amplified using the primers indicated in Table S2 and cloned by Gibson assembly (48). The plasmids were introduced into the B. subtilis conjugation strain JAB980 (35) by natural transformation (50). The resulting strains were then used to introduce the modified ICEBs1 element into the appropriate B. thuringiensis strains by conjugation as previously described (35). Briefly, donor strains were grown in LB plus Cam plus d-Ala to mid-log phase, and then expression of *rapI* (RapI induces conjugation) was induced by incubation with xylose for 1 h. The recipients were also grown to mid-log phase, mixed at equal volumes, plated on LB plus d-Ala, and incubated for 4 h. Cells were collected from the plates and spread onto LB plus Cam without d-Ala to select for transconjugants.

### β-Galactosidase assays.

To quantify expression from different promoters, we measured the levels of *lacZ* promoter fusions in β-galactosidase assays. Overnight cultures (10 μL) were spotted onto LB–X-Gal plates lacking or containing cefoxitin (5 μg/mL) and incubated overnight at room temperature. Bacteria from each patch were scraped from the plates and resuspended in 1 mL Z-buffer. Cells were permeabilized by mixing with chloroform and 2% Sarkosyl (51, 52). Permeabilized cells (150 μL) were mixed with 10 mg/mL *ortho*-nitrophenyl-β-galactoside (ONPG; 50 μL; RPI Corp.). OD_600_ was measured once at the beginning of the assay to determine the cell density of each sample. OD_405_ was measured over time using an Infinite M200 Pro (Tecan) every 2 min. β-Galactosidase activity units, determined by the formula micromoles of ONP formed per minute × 10^3^/OD_600_ × volume (in milliliters) of cell suspension, were calculated as previously described (53). Experiments were performed in technical triplicate, with the mean and standard deviation shown. Each experiment was performed in biological triplicate, and representative data are presented.

### MIC assay.

To determine the MICs of various antibiotics, we diluted bacteria grown overnight (washed in LB) 1:500 in media containing dilutions of each antibiotic. All MIC experiments were performed in flat-bottomed 96-well plates. Each experiment was performed in technical triplicate, and incubation for 24 h was allowed before observation for growth or no growth. Each experiment was performed in biological triplicate, and representative data are presented.

### Nitrocefin assays.

Nitrocefin (Sigma) was resuspended in dimethyl sulfoxide (DMSO; 5.16 mg/mL) and then diluted 1:20 in 0.1 M phosphate buffer (pH 7). Equal volumes of diluted nitrocefin were added to culture supernatants. Absorbance at a wavelength of 490 nm was measured using an Infinite M200 Pro plate reader (Tecan) every 30 s for 5 min. The increase in *A*_490_ over time was calculated.
