# Supplementary material for: Identification of the Extracytoplasmic Function σ Factor σP Regulon in Bacillus thuringiensis
Source: mSphere. 2022 Jan 26;7(1):e00967-21. doi: 10.1128/msphere.00967-21 (PMC8791391; doi:10.1128/msphere.00967-21)
Supplement: TABLE S1 [file msphere.00967-21-st001.pdf]

TABLE S1. Plasmids used in this study

Table S1. Plasmids

Plasmid	Relevant features	Parent vector	Restriction enzymes to digest parent vector	PCR primers	PCR template	Reference
pTHE950	pE194ts, *'thrC lacZ thrB'*					23
pTHE954	pE194ts, *'thrC* P_{pbpM} *-lacZ thrB'*	pTHE950	XhoI, SacI	2931-32	AW43	This study
pTHE955	pE194ts, *'thrC* P_{pbpP} *-lacZ thrB'*	pTHE950	XhoI, SacI	2933-34	AW43	This study
pTHE951	pE194ts, *'thrC* P_{pbpN} *-lacZ thrB'*	pTHE950	XhoI, SacI	2926-27	AW43	This study
pBT7	pE194ts, *'thrC* P_{dacC} *-lacZ thrB'*	pTHE950	XhoI, SacI	2941-42	AW43	This study
pBT9	pE194ts, *'thrC* P_{bla1} *-lacZ thrB'*	pTHE950	XhoI, SacI	2935-36	AW43	This study
pBT8	pE194ts, *'thrC* P_{bla2} *-lacZ thrB'*	pTHE950	XhoI, SacI	2973-74	AW43	This study
pTHE956	pE194ts, *'thrC* P_{bla3} *-lacZ thrB'*	pTHE950	XhoI, SacI	2943-44	AW43	This study
pTHE957	pE194ts, *'thrC* P_{bla4} *-lacZ thrB'*	pTHE950	XhoI, SacI	2975-76	AW43	This study
pTHE958	pE194ts, *'thrC* P_{bt2718} *-lacZ thrB'*	pTHE950	XhoI, SacI	2977-78	AW43	This study
pTHE959	pE194ts, *'thrC* P_{bt1021} *-lacZ thrB'*	pTHE950	XhoI, SacI	2979-80	AW43	This study
pCE801	pE194ts, *'thrC* P_{dltX} *-lacZ thrB'*	pTHE950	XhoI, SacI	5028-29	AW43	This study
pCE802	pE194ts, *'thrC* P_{dltC} *-lacZ thrB'*	pTHE950	XhoI, SacI	5030-31	AW43	This study
pCE803	pE194ts, *'thrC* P_{bt0552} *-lacZ thrB'*	pTHE950	XhoI, SacI	5032-33	AW43	This study
pCE697	ICE*Bs1*::P_{IPTG} *amp cat*					24
pCE727	ICE*Bs1*::P_{IPTG} *-bla1 amp cat*	pJAB980	SalI, NheI	4662-63	AW43	This study
pCE809	ICE*Bs1*::P_{IPTG} *-bla2 amp cat*	pJAB980	SalI, NheI	5024-25	AW43	This study
pCE810	ICE*Bs1*::P_{IPTG} *-bla3 amp cat*	pJAB980	SalI, NheI	5052-53	AW43	This study
pCE808	ICE*Bs1*::P_{IPTG} *-bla4 amp cat*	pJAB980	SalI, NheI	5026-27	AW43	This study
pCE699	ICE*Bs1*::P_{IPTG} *-pbpM amp cat*	pJAB980	SalI, NheI	4519-20	AW43	This study
pCE708	ICE*Bs1*::P_{IPTG} *-pbpN amp cat*	pJAB980	SalI, NheI	4517-18	AW43	This study
pCE729	ICE*Bs1*::P_{IPTG} *-dacC amp cat*	pJAB980	SalI, NheI	4666-67	AW43	This study
pCE821	ICE*Bs1*::P_{IPTG} *-bt2718 amp cat*	pJAB980	SalI, NheI	5126-17	AW43	This study
pMAD	ori-pE194ts					49
pBT1	ori-pE194ts, Δ*pbpM*	pMAD	EcoRI, BglII	3828-29; 3830-31	AW43	This study
pBT3	ori-pE194ts, Δ*pbpN*	pMAD	EcoRI, BglII	3820-21; 3822-23	AW43	This study
pBT14	ori-pE194ts, Δ*dacC*	pMAD	EcoRI, BglII	2948-49; 2950-51	AW43	This study
pCE792	ori-pE194ts, Δ*bla2*	pMAD	EcoRI, BglII	2981-82; 2983-84	AW43	This study
pCE826	ori-pE194ts, Δ*bla3*	pMAD	EcoRI, BglII	5133-34; 5135-36	AW43	This study
pCE793	ori-pE194ts, Δ*bla4*	pMAD	EcoRI, BglII	2985-86; 2987-88	AW43	This study
pCE820	ori-pE194ts, Δ*bt2718*	pMAD	EcoRI, BglII	5105-06; 5107-08	AW43	This study
pEBT4	ori-pE194ts, Δ*bla1*	pMAD	EcoRI, BglII		AW43	23
