# Supplementary material for: Identification of the Extracytoplasmic Function σ Factor σP Regulon in Bacillus thuringiensis
Source: mSphere. 2022 Jan 26;7(1):e00967-21. doi: 10.1128/msphere.00967-21 (PMC8791391; doi:10.1128/msphere.00967-21)
Supplement: TABLE S2 [file msphere.00967-21-st002.pdf]

TABLE S2. Primers used in this study

Table S2. Primers

Oligo	Sequence	Relevant features
2931	GCAATGCCCGGTTTTTTTTCTCGAGATATAACCCTCCCTTATGTAG	Clone P_{pbpM}-*lacZ* fusion
2932	TCAACAAGCTGGGGATCCGCGGCCGCAAATCCTCCATTTAATCCTTTC	Clone P_{pbpM}-*lacZ* fusion
2933	GCAATGCCCGGTTTTTTTTCTCGAGAAATCCTCCATTTAATCCTTTC	Clone P_{pbpP}-*lacZ* fusion
2934	TCAACAAGCTGGGGATCCGCGGCCGCATATAACCCTCCCTTATGTAG	Clone P_{pbpP}-*lacZ* fusion
2926	CAATGCCCGGTTTTTTTTCTCGAGAATTATTAAAAATAGAACAAATACATGATTTTTATC	Clone P_{pbpN}-*lacZ* fusion
2927	TCAACAAGCTGGGGATCCGCGGCCGCAGCATCCATAATTTTTTCAATTCAATCTTC	Clone P_{pbpN}-*lacZ* fusion
2941	GCAATGCCCGGTTTTTTTTCTCGAGATCAGTTAGAAAAACTACATCCAG	Clone P_{dacC}-*lacZ* fusion
2942	TCAACAAGCTGGGGATCCGCGGCCGCTTGCAAAACATACCTTTCAC	Clone P_{dacC}-*lacZ* fusion
2935	GCAATGCCCGGTTTTTTTTCTCGAGTAGAATACTTTTCTCAGGGAAG	Clone P_{bla1}-*lacZ* fusion
2936	TCAACAAGCTGGGGATCCGCGGCCGCTTCCTTCCAAATTTTTAGCAC	Clone P_{bla1}-*lacZ* fusion
2973	TGCCCGGTTTTTTTTCTCGAGTTTTTTCCTTACTAGAAGCG	Clone P_{bla2}-*lacZ* fusion
2974	TCAACAAGCTGGGGATCCGCGTCATCAACCCTTTCCTTCA	Clone P_{bla2}-*lacZ* fusion
2943	GCAATGCCCGGTTTTTTTTCTCGAGGAAAAATTAAATTTATTTAATCTTATTCGTTTAAC	Clone P_{bla3}-*lacZ* fusion
2944	TCAACAAGCTGGGGATCCGCGGCCGCAACTTGTTTAAGACTATCAT	Clone P_{bla3}-*lacZ* fusion
2975	TGCCCGGTTTTTTTTCTCGAATTTTATAGGATACAACTAAATATTAGT	Clone P_{bla4}-*lacZ* fusion
2976	TCAACAAGCTGGGGATCCGCATGTATTCCCCCTTTATTTTT	Clone P_{bla4}-*lacZ* fusion
2977	TGCCCGGTTTTTTTTCTCGAATTTTATAGGATACAACTAAATATTAGT	Clone P_{bt2718}-*lacZ* fusion
2978	TCAACAAGCTGGGGATCCGCATGTATTCCCCCTTTATTTTT	Clone P_{bt2718}-*lacZ* fusion
2979	TGCCCGGTTTTTTTTCTCGAATTTCATAGATAATAGAGAGCTG	Clone P_{bt1012}-*lacZ* fusion
2980	TCAACAAGCTGGGGATCCGCGAAAAATAAATCTCCTCTCAAA	Clone P_{bt1012}-*lacZ* fusion
5030	TGCCCGGTTTTTTTTCTCGACTTTTTCATGAAATGATAATTCCGG	Clone P_{dltX}-*lacZ* fusion
5031	TCAACAAGCTGGGGATCCGCCCCTCTCTTAATGAAATAATAGCTC	Clone P_{dltX}-*lacZ* fusion
5032	TGCCCGGTTTTTTTTCTCGAGCAACGAAGAAAAAGTTAATTAAGA	Clone P_{dltC}-*lacZ* fusion
5033	TCAACAAGCTGGGGATCCGCCTTGCTCTTTGAATTCTGCC	Clone P_{dltC}-*lacZ* fusion
4662	TAACAATTAAGCTTAGTCGATAAAAAGGTGCTAAAAATTTGGAA	Clone P_{IPTG}-*bla1*
4663	ATTAGCTTGCATGCGGCTAGTTATCTAAGAGCCTTAACTATAACTTTAG	Clone P_{IPTG}-*bla1*
5024	TAACAATTAAGCTTAGTCGAGGCTTGAAGGAAAGGGTTGA	Clone P_{IPTG}-*bla2*
5025	ATTAGCTTGCATGCGGCTAGACAATTTCTTATTTTAATAAATCCAATGTAT	Clone P_{IPTG}-*bla2*
5052	TAACAATTAAGCTTAGTCGACTAAAAAACATATATTCTTGAAAGAGGT	Clone P_{IPTG}-*bla3*
5053	ATTAGCTTGCATGCGGCTAGTTATTTATTTGTCACTTTTAAAGCATCT	Clone P_{IPTG}-*bla3*
5026	TAACAATTAAGCTTAGTCGATTGAAAGAGGTTGAAAATTCATG	Clone P_{IPTG}-*bla4*
5027	ATTAGCTTGCATGCGGCTAGCTATTAATTTATTAGTTTTTATTCGTAACTTTTAA	Clone P_{IPTG}-*bla4*
4519	TAACAATTAAGCTTAGTCGACGATTAAATGGAGGATTTATGAAAGAACA	Clone P_{IPTG}-*pbpM*
4520	ATTAGCTTGCATGCGGCTAGCCCTGCTGGTTTATATGGCGCG	Clone P_{IPTG}-*pbpM*
4517	TAACAATTAAGCTTAGTCGAC CATCATTTTTAACTAAGGGAGAAGA	Clone P_{IPTG}-*pbpN*
4518	ATTAGCTTGCATGCGGCTAGC ACCATATAATTAAGTATTATTTCTGAAATACA	Clone P_{IPTG}-*pbpN*
4666	TAACAATTAAGCTTAGTCGACTAATATTTAGTTGTATCCTATAAAATTAGCT	Clone P_{IPTG}-*dacC*
4667	ATTAGCTTGCATGCGGCTAGTGTTTTGCAAGAGAGTTATTGC	Clone P_{IPTG}-*dacC*
5126	TAACAATTAAGCTTAGTCGAGTATAGTATAGATATGTTTAAAAATAAAGGGG	Clone P_{IPTG}-*bt2718*
5127	ATTAGCTTGCATGCGGCTAGTATTCGTTACATTGCAAACTATTATT	Clone P_{IPTG}-*bt2718*
3828	ACACATTAACTAGACAGATCACTTTTTGAATGTCCCATAAGAA	D*pbpM*
3829	TTCCTGTCTTTCCTGCTGGTAAATCCTCCATTTAATCCTTTCAG	D*pbpM*
3830	AAGGATTAAATGGAGGATTTACCAGCAGGAAAGACAGGAA	D*pbpM*
3831	CTGCAGAAGCTTCTAGAATTCTTTAGGTCCGCCTATTTTATT	D*pbpM*
3820	ACACATTAACTAGACAGATCTAGCCATCAAGGAACACAGG	D*pbpN*
3821	ATTCACCATATAATTAAGTACCATAATTTTTTCAATTCAATCTTCTCCC	D*pbpN*
3822	GAAGATTGAATTGAAAAAATTATGGTACTTAATTATATGGTGAATTTTTTGACT	D*pbpN*
3823	CTGCAGAAGCTTCTAGAATTCTTCATCTTTACGATTCTCCATT	D*pbpN*
2981	ACACATTAACTAGACAGATCTTAGTAATTAAGAAAAAAGATCATTAT	D*bla2*
2982	TTTTGTATTTCTACAATTTCGTCATCAACCCTTTCCTTCA	D*bla2*
2983	TGAAGGAAAGGGTTGATGACGAAATTGTAGAAATACAAAAGAGA	D*bla2*
2984	CTGCAGAAGCTTCTAGAATTTCCCCACTTTCATAGCCATA	D*bla2*
5133	ACACATTAACTAGACAGATCGCCTGCTTTATTAGTATATTCTCT	D*bla3*
5134	CGGGAAAATAATAATAACTAGTTACTATTACATTTTCAACCTCTTTCAAGAATAT	D*bla3*
5135	CTTGAAAGAGGTTGAAAATGTAATAGTAACTAGTTATTATTATTTTCCCGG	D*bla3*
5136	CTGCAGAAGCTTCTAGAATTGAAATTGGAATTATTGAATAAAAAAGTGT	D*bla3*
2985	ACACATTAACTAGACAGATCTATTTAAGATAATCTCATATAAATTACTT	D*bla4*
2986	CACGGAAAACTATTAATTTAGAATTTTCAACCTCTTTCAAC	D*bla4*
2987	TTGAAAGAGGTTGAAAATTCTAAATTAATAGTTTTCCGTGGT	D*bla4*
2988	CTGCAGAAGCTTCTAGAATTTGTCTAGATGATATTTTTTGAAG	D*bla4*
5105	ACACATTAACTAGACAGATCGAACTTTAACTGGATGTTAAAAGG	D*bt2718*
5106	ATTCGTTACATTGCAAACTAATGTATTCCCCCTTTATTTTTAAACA	D*bt2718*
5107	AAAATAAAGGGGGAATACATTAGTTTGCAATGTAACGAATATGA	D*bt2718*
5108	CTGCAGAAGCTTCTAGAATTAATTAATCATTATTGACATCTCACCT	D*bt2718*
2948	GGTACCCGGGAGCTCGAATTGAAGCGAGGAATAAAGTTAT	D*dacC*
2949	TAGTTGTATCCTATAAAATTTACCTTTCACTTCTGTTACC	D*dacC*
2950	GGTAACAGAAGTGAAAGGTAAATTTTATAGGATACAACTAAATATTAG	D*dacC*
2951	GCGTCGGGCGATATCGGATCATTATTTTGTCTTCTGTACAGG	D*dacC*
